# Hierarchical VO*_x_*@Wood Aerogel Electrodes with Tunable Valence States for Enhanced Energy Storage

**DOI:** 10.3390/nano15161249

**Published:** 2025-08-14

**Authors:** Yu Wang, Yuan Yu, Zhenle Hu, Lei Qiao, Huaiyuan Peng, Jingwen Xie, Haiyue Yang, Chengyu Wang

**Affiliations:** Key Laboratory of Bio-Based Material Science and Technology of Ministry of Education, Northeast Forestry University, Harbin 150040, China; yuwang@nefu.edu.cn (Y.W.); dlyy@nefu.edu.cn (Y.Y.); huzhenle@nefu.edu.cn (Z.H.); 2023111282@nefu.edu.cn (L.Q.); 2021213591@nefu.edu.cn (H.P.); wyl_2022@nefu.edu.cn (J.X.)

**Keywords:** vanadium oxide, wood, porous materials, mixed-valence, electrochemical energy storage

## Abstract

Vanadium-based electrode materials are limited in practical applications, due to their low energy density, cycling instability, and poor electrochemical stability. To address these limitations, a wood-derived vanadium oxide (VO*_x_*) electrode was developed through sol–gel assembly followed by thermal annealing, in which VO*_x_* aerogel formed within the vertically aligned wood channels, resulting in a continuous porous network to mitigate particle aggregation and enhance ion diffusion. After thermal annealing at 800 °C, V^5+^ partially converts to V^4+^, forming a mixed-valence heterostructure that significantly increases the density of redox-active sites and facilitates efficient charge transfer. The optimized VO*_x_*@Wood-800 °C (VOW-800) electrode exhibits a high specific capacitance of 317.8 F g^−1^ at 2 mA cm^−2^ and a specific surface area of 111.22 m^−2^ g^−1^, attributed to the synergistic effects of the mixed-valence structure and the enhanced ion accessibility provided by the wood-derived porous framework. This approach offers a promising pathway for developing vanadium-based electrodes with improved charge storage capacity and interface stability.

## 1. Introduction

Modern electronic devices and sustainable energy systems have a growing need for reliable energy storage, necessitating the development of advanced energy storage technologies [1,2,3,4]. Supercapacitors, known for their fast charge–discharge kinetics and long cycle life, represent a promising solution bridging the gap between conventional capacitors and batteries [5,6,7]. However, there are challenges of achieving high energy density and long-term stability in supercapacitors, due to its insufficient ion transport, limited active surface area, and structural instability during cycling of conventional electrodes [8,9,10].

Vanadium oxides (VO*_x_*) have attracted significant attention as electrode materials owing to their rich redox chemistry, high theoretical capacitance, and relative abundance. Despite these advantages, practical applications are hindered by poor electrical conductivity and severe particle agglomeration, which limit electron mobility and compromise electrochemical stability [11,12]. Integrating VO*_x_* into porous frameworks enhances ion diffusion and exposes more active sites, offering a viable strategy to mitigate these limitations [13,14,15]. When V_2_O_5_ is integrated with graphene aerogel, the composite achieves a specific capacitance of 204 F g^−1^ at 1 A g^−1^ [16,17], representing 35% improvement over pure V_2_O_5_ nanowires (158 F g^−1^ at the same current density). Nevertheless, conventional methods such as direct impregnation or chemical vapor deposition often result in non-uniform coverage and weak interfacial contact [18,19,20]. Achieving a uniform distribution and firm bonding between VO*_x_* and porous substrates still remains challenging and requires innovative synthesis strategies [21,22,23].

Natural wood emerges as an ideal scaffold with inherent oxygen-containing functional groups (-OH, -COOH) and a 3D porous architecture that enables the strong chemical anchoring of vanadium species through coordination bonds while facilitating efficient mass transport [24,25]. Crucially, the carbonization process not only preserves this structural advantage but also induces the formation of mixed-valence vanadium species (V^4+^/V^5+^) [26,27]. This valence engineering creates electron-deficient V^5+^ and electron-rich V^4+^ sites that synergistically enhance charge storage through complementary redox reactions: V^5+^ + e^−^ ⇄ V^4+^ and V^4+^ + e^−^ ⇄ V^3+^ [28]. The resulting heterostructure significantly increases charge carrier density while improving electrical conductivity through inter-valence charge transfer mechanisms [29,30]. Consequently, wood-derived hierarchical porous carbon/vanadium oxide composites exhibit superior capacitive performance compared to freestanding V_2_O_5_ electrodes (185 F g^−1^) [31], owing to synergistic effects between the conductive carbon framework and redox-active multivalent vanadium species. These unique characteristics position wood-derived composites as promising candidates for overcoming the intrinsic limitations of conventional vanadium oxide electrodes.

Herein, a sol–gel-assisted infiltration approach was employed to construct VO*_x_* networks within wood channels, followed by nitrogen-assisted pyrolysis to achieve template carbonization and valence modulation. This strategy enables the formation of V_2_O_5_ nanowire aerogels within the aligned wood structure, which are then converted into mixed-valence VO*_x_* heterostructures (V^4+^/V^5+^) through controlled thermal treatment. The VO*_x_* aerogel electrodes (VO*_x_*@Wood) with interconnected porous structure, enhance ion diffusion and charge transport. VO*_x_*@Wood treated at 800 °C (VOW-800) exhibits a specific surface area of 111.22 m^2^ g^−1^. As a result, the VOW-800 exhibits a high specific capacitance of 317.8 F g^−1^ at 2 mA cm^−2^ while maintaining excellent structural stability during cycling. These findings provide a sustainable pathway for developing high-performance vanadium-based supercapacitors, overcoming traditional challenges related to poor conductivity and particle agglomeration in transition metal oxide electrodes. This hierarchical integration strategy effectively overcomes the inherent defects of vanadium oxide electrodes and addresses traditional challenges such as poor conductivity and particle agglomeration in transition metal oxide electrodes, providing a feasible solution for the development of high-performance vanadium-based supercapacitors.

## 2. Materials and Methods

### 2.1. Materials

Balsa wood (BW) was cut precisely into dimensions of 10.0 × 10.0 × 1.0 mm^3^, maintaining a vertical section aligned with the natural growth direction of the wood to preserve structural integrity. Ammonium metavanadate (NH_4_VO_3_, 99%) and potassium chloride (KCl) were obtained from Shanghai Aladdin Biochemical Technology Co. Ltd. (No. 809, Chuhua Branch Road, Fengxian District, Shanghai, China), while potassium hydroxide (KOH) was supplied by Sinopharm Chemical Reagent Co. Ltd. (No. 801, Huta Road, Jing’an District, Shanghai, China). All chemicals were used as received without further purification for subsequent experimental procedures.

### 2.2. Preparation of V_2_O_5_@Wood

Firstly, 2.0 g of ammonium metavanadate (NH_4_VO_3_) was dispersed in 2 mL of deionized water with continuous grinding and stirring for 3 min to ensure uniform mixing. The resulting mixture was then transferred into a beaker containing 20 mL of 1 mol L^−1^ hydrochloric acid (HCl) and stirred thoroughly to promote dissolution. The supernatant was carefully decanted, and 40 mL of deionized water preheated to above 80 °C was added. After stirring and allowing it to stand until the precipitation ceased, the supernatant was removed. The hot water rinsing step is repeated multiple times to ensure the complete removal of residual ions. Subsequently, 40 mL of hot deionised water (temperature exceeding 80 °C) is added again, and the mixture is allowed to stand for several days to promote equilibrium. The purpose of aging for several days is to ensure more thorough mixing of the solution, allowing more V oxides to dissolve into the liquid and form hydrates. Subsequently, BW is immersed in the solution and subjected to three vacuum degassing cycles using a vacuum oven to ensure the solution penetrates deeply into the porous wood structure. The formed V_2_O_5_@Wood is gently separated from the aqueous solution and immersed in a 1 mol L^−1^ KCl solution for ion exchange while wet. Initially, the nanowires dispersed in the dispersion liquid do not come into contact with each other under the influence of intermolecular forces, thereby maintaining a dynamic equilibrium, while the dispersion liquid exhibits sol–gel characteristics. When the hydrated vanadium pentoxide dispersion comes into contact with a metal cation (K^+^) solution, gelation occurs at the contact site. This mechanism can be explained by the adsorption of opposite charges. The surface of V_2_O_5_ nanowires carries a surface charge, typically with a negative charge on the particle surface. Upon contact with metal cations, the charges adsorb onto the particle surfaces. Under the influence of these charges, V_2_O_5_ nanoparticles aggregate to form a gel, disrupting the overall stability of the dispersion liquid. A static nanowire network begins to form, with K^+^ diffusing from the solution to the interface between the dispersion liquid and the gel, causing the gel-like volume in the dispersion liquid to continuously increase, ultimately transforming all the sol into gel. At this point, the V_2_O_5_ hydrates contained within BW undergo complete ion exchange, forming a visually observable orange-red gel-like substance both internally and on the surface. After soaking, the V_2_O_5_@Wood is removed, and the KCl solution is poured into the previously obtained red filtrate to convert the V-containing liquid into orange-red gel-like substance for waste disposal (including the V_2_O_5_ nanowires released during synthesis according to the preparation procedure described in Reference [32]). The gelled fraction was discarded, and the remaining portion was subjected to freeze-drying, yielding the final V_2_O_5_ aerogel@wood composite.

### 2.3. Preparation of VO_x_@Wood

VO*_x_* aerogel@wood electrodes (VOW) were prepared by pyrolysing V_2_O_5_@BW at 600 °C, 700 °C, 800 °C, and 900 °C with heating rate of 5 °C min^−1^ for three hours under nitrogen atmosphere, respectively.

## 3. Results and Discussion

### 3.1. Microstructural Characterization of VOx@Wood at Different Temperatures

**Figure 1:**
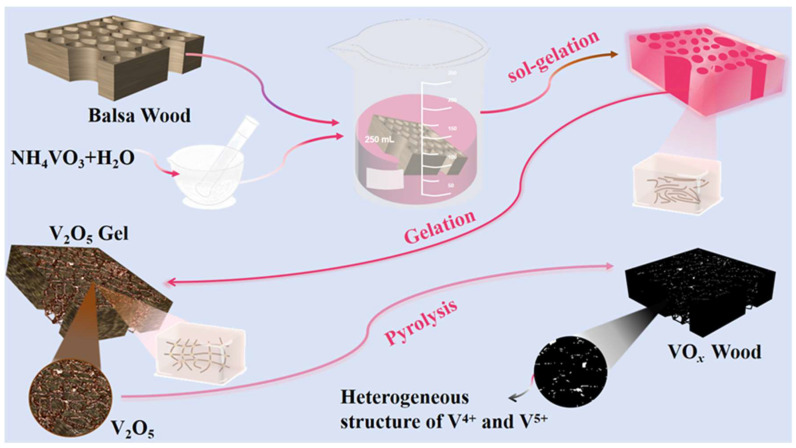
Schematic diagram of the preparation process of VOW.

BW contains multiple hollow and longitudinal channels with diameters exceeding 45 μm (Figures S1 and S2). These inherent porous structures provide an ideal scaffold for the infiltration and anchoring of VO*_x_*, enabling the colloidal V_2_O_5_ to penetrate the hollow channels and form a uniform V_2_O_5_ layer (Figure 1). After freeze-drying, the colloidal V_2_O_5_ transformed into a stable gel, forming a robust network that adhered firmly to the inner surfaces of the wood channels (Figure S3). Subsequently, the V_2_O_5_@Wood composite was subjected to thermal treatment to induce carbonization and transform the V_2_O_5_ into mixed-valence VO*_x_*.

**Figure 2:**
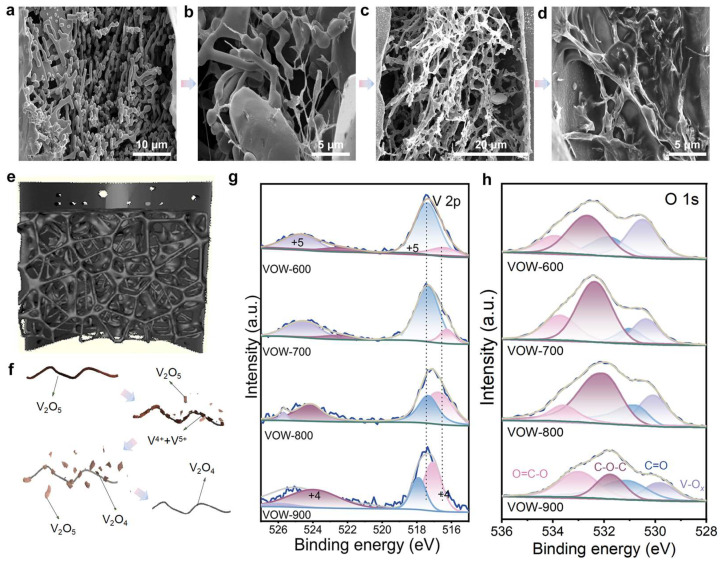
Microstructural and spectroscopic properties of VO*_x_*. The SEM images of (**a**) VOW-600, (**b**) VOW-700, (**c**) VOW-800, and (**d**) VOW-900; (**e**) 3D illustration of the internal pore structure of VOW-800, (**f**) 3D illustration of the V_2_O_5_ cleavage process, (**g**) XPS spectrum of V 2p, and (**h**) O 1s for VOW.

To investigate the effects of thermal treatment, the composite samples were heated at various temperatures ranging from 600 °C to 900 °C. Scanning electron microscopy (SEM) was used to observe the morphological changes of VO*_x_* under different thermal conditions. There are rod-like VO*_x_* nanowires formed from the V_2_O_5_/KCl precursor solution at 600 °C (Figure 2a). When the temperature was increased to 700 °C, the structure began to fragment, with some of the nanowire network partially collapsing (Figure 2b). At 800 °C, a significant transformation occurred, forming an interconnected filamentous network resembling an aerogel (Figure 2c). However, further increasing the temperature to 900 °C, the porous network collapsed, forming dense VO*_x_* aggregates with compromised integrity. This phenomenon can be primarily attributed to thermal-induced sintering and structural reconstruction. Under high-temperature conditions, VO*_x_* nanoparticles exhibit enhanced atomic mobility, which promotes particle coalescence and densification [33,34,35]. As a result, the high surface area and porous architecture are gradually lost due to surface energy minimization, leading to the formation of compact bulk structures [36,37,38]. Additionally, the elevated thermal energy may trigger phase transformations or reduction reactions between different vanadium oxidation states (V^5+^ to V^4+^), further disrupting the stability of the original nanostructure [39,40,41]. These combined effects ultimately result in the loss of the porous framework and the formation of dense VO*_x_* agglomerates (Figure 2d) [42,43].

The 3D schematic illustration reveals the internal structure of thermally treated wood pores. At 600 °C, V_2_O_5_ exists as nanowires, which are predominantly confined within the wood pores (Figure S4). When the temperature is increased to 700 °C, the outer layers of the nanowires begin to fracture and peel off, accompanied by partial cross-linking, forming a localized network structure (Figure S5). Upon further heating to 800 °C, the majority of the V_2_O_5_ nanowires are exfoliated and reorganized into an interconnected VO*_x_* mesh structure in both longitudinally and transversely of the wood channels. This transition results in a significant increase in specific surface area and a notable improvement in pore space utilization (Figure 2e). However, at 900 °C, extensive breakage of the cross-links occurs, causing the collapse of the porous network (Figure S6). A 3D schematic of the V_2_O_5_ cracking process is shown in Figure 2f. To characterize the lattice characteristics of the electrode material, X-ray diffraction (XRD) analysis was performed. The characteristic peaks at (003), (111), and (001) correspond to the V^4+^structure, and the diffraction intensity of these crystal planes decreases gradually with increasing temperature, indicating a gradual decrease in V^5+^ content. The characteristic peaks at (101), (210), (211), (002), and (221) correspond to the V^4+^ structure, vanadium oxide begins to decompose at 600 °C, and the diffraction intensity of its crystal planes increases continuously with rising temperature, indicating that the higher the temperature, the greater the V^4+^ content. At 800 °C, the characteristic peak intensities of the two valence states are similar and form a strong heterostructure with V^5+^, which can be linked to form a heterostructure. At other treatment temperatures, the characteristic peak intensities of the two valence states differ significantly, making it difficult to form a stable network structure (Figure S7).

The oxidation states of vanadium in the VO*_x_*@wood composites were investigated using X-ray photoelectron spectroscopy (XPS). The V 2p spectra reveal a temperature-dependent change in the vanadium oxidation state (Figure 2g). At 600 °C, a distinct V 2p peak emerged at 517.48 eV, characteristic of vanadium in the V^5+^ [44]. As the annealing temperature increased to 700 °C, the V 2p binding energy shifted and the spectrum exhibited peak splitting. Subsequent peak fitting analysis revealed a gradual reduction in V^5+^ species and a concurrent increase in V^4+^ content, indicating a partial valence transition consistent with thermal reduction mechanisms observed in vanadium oxide systems [45]. At 800 °C, the main peak further shifted to 517.18 eV, where a coexisting mixed-valence state of V^4+^ and V^5+^ was established, suggesting the formation of a heterogeneous vanadium oxide structure with enhanced electrochemical activity [46]. Upon further annealing to 900 °C, the V 2p peak shifted to 516.58 eV, implying near-complete reduction to V^4+^, with minimal residual V^5+^ detected, a phenomenon associated with excessive thermal treatment that compromises structural stability [47]. The VOW-800 specifically exhibits both V^5+^ and V^4+^ species, demonstrating the formation of a heterogeneous VO*_x_* phase with multiple oxidation states. This mixed-valence configuration enhances redox activity and promotes efficient charge transfer, which are essential for the superior capacitive performance observed in VOW-800.

High-resolution XPS analysis of the O 1 s spectra further reveals the surface chemistry of the composites. The spectra show distinct peaks corresponding to oxygen-containing functional groups, including O=C-O, C-O-C, C=O, and V-O*_x_* bonds, present in all samples. The high-resolution O 1 s XPS spectra reveal the temperature-dependent evolution of oxygen-containing functional groups and vanadium oxide species in the VOW samples. At 600 °C, the broad fitted peaks are mainly attributed to organic oxygen functionalities such as C-O-C and O=C-O, indicating incomplete carbonization of the wood-derived precursor [48]. The weak V-O*_x_* signal suggests that the vanadium species remain largely unoxidized at this stage. Upon annealing at 700 °C, the V-O*_x_* component becomes more pronounced, reflecting the onset of vanadium oxide formation [49]. When the temperature increases to 800 °C, the V-O*_x_* signal is significantly intensified, suggesting the construction of a V-O*_x_* heterostructure, while certain oxygen-containing organic groups are still retained. These residual functional groups are believed to promote the development of a conductive carbon framework through defect-mediated charge transfer pathways. However, at 900 °C, the intensities of both C-O-C and V-O*_x_* components are markedly reduced, indicating that the organic species have been transformed into a carbon matrix and the heterostructure has collapsed due to over-oxidation or excessive thermal treatment [50]. The coexistence of these oxygen functionalities contributes to the robust interfacial bonding and improved electronic conductivity, which collectively support the high performance of the VOW-800 electrode (Figure 2h).

### 3.2. Performance Testing of VO_x_@Wood at Different Temperatures

**Figure 3:**
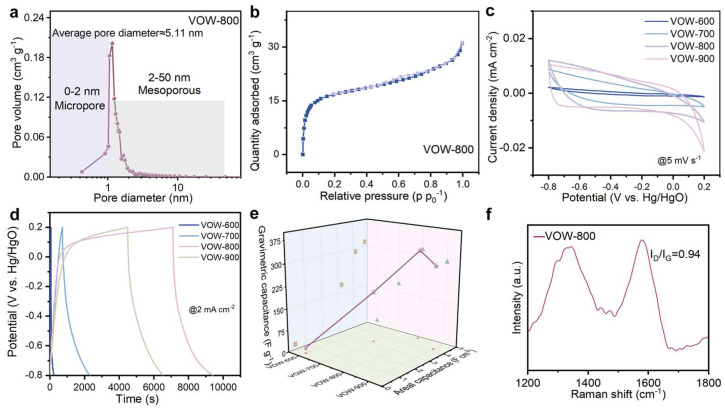
The pore structure of VOW at different temperatures. (**a**) Pore size distributions of VOW-800, (**b**) N_2_ adsorption/desorption curves of VOW-800, (**c**) cyclic voltammetry (CV) curves of VOW at a scan rate of 5 mV s^−1^, (**d**) GCD curves of VOW with a current density of 2 mA cm^−2^, (**e**) areal specific capacitance and gravimetric capacitance of VOW at different temperatures, (**f**) Raman spectra of VOW-800.

The thermal pyrolysis process not only facilitates the partial reduction of V_2_O_5_ into multi-valence vanadium oxides but also converts the wood scaffold into a porous carbon matrix. This dual transformation results in a hierarchical porous structure composed of interconnected micro- and mesopores, which enhances ion transport and charge storage capacity. Brunauer–Emmett–Teller (BET) analysis confirms the well-developed porosity of the VOW-800 composite, with an average pore diameter of 5.11 nm, reflecting its rich micro-mesoporous structure (Figure 3a). The N_2_ adsorption–desorption isotherms exhibit a sharp increase in adsorption volume at high relative pressures (P/P_0_ ≈ 1), indicative of capillary condensation within mesopores and the presence of interconnected pore networks. The specific surface area of VOW-800 reaches 111.22 m^2^ g^−1^, which is significantly higher than that of pristine balsa wood and samples treated at other temperatures (Figure 3b and Figures S8–S15, Table S1). This result indicates that VO*_x_* not only serves as a structural component but also actively contributes to the formation of a micro-/nanostructured system at 800 °C that substantially improves the overall porosity and structural functionality of the composite. Compared to pristine BW, the resulting porous framework exhibits a higher pore density and enhanced connectivity, providing more active sites.

To evaluate the electrochemical performance of VOW electrodes obtained at different thermal treatment temperatures, cyclic voltammetry (CV) measurements were conducted at a scan rate of 5 mV s^−1^ in 6.0 M KOH [51,52]. The CV curves of VOW-800 and VOW-900 exhibit larger enclosed areas compared to those of VOW-600 and VOW-700, indicating enhanced charge storage capacity, likely due to improved ion accessibility and abundant redox-active sites. In contrast, the CV profiles of VOW-600 and VOW-700 are relatively narrow and lack prominent redox features, reflecting their lower charge storage capacity due to inadequate thermal activation, which fails to induce significant alterations in the vanadium oxidation states. As a result, the formation of a robust three-dimensional network structure within the hierarchical wood-derived porous matrix is hindered, which leads to compromised electrochemical performance (Figure 3c). To further investigate the charge-storage capacity, galvanostatic charge–discharge (GCD) tests were performed at a current density of 2 mA cm^−2^ (Figure 3d). The GCD curves demonstrate that VOW-800 exhibits the longest charge–discharge duration among the samples, indicating superior energy storage capacity. The relatively linear and symmetric GCD profiles suggest good electrochemical reversibility and low internal resistance. This observation is further supported by the EIS analysis, where the Nyquist plot (Figure S16) shows a low charge transfer resistance (Rct) of 12.6 Ω. Such an Rct value signifies efficient electron transport, corroborating the stable charge–discharge behavior observed in GCD tests. The extended discharge time can be attributed to the optimized porous carbon network and the presence of mixed-valence vanadium oxides (V^4+^/V^5+^) formed during thermal treatment at 800 °C. In contrast, the reduced discharge durations exhibited by VOW-600, VOW-700, and VOW-900 are primarily due to the incomplete development of the carbonaceous network and the limited pore structure induced by lower or excessive thermal treatment temperatures. The specific capacitance of the VOW electrodes was calculated from the GCD curves at different current densities. At a current density of 2 mA cm^−2^, VOW-800 achieves an areal capacitance of 4.82 F cm^−2^, which is significantly higher than that of VOW-900 (4.18 F cm^−2^), VOW-700 (3.24 F cm^−2^), and VOW-600 (0.38 F cm^−2^). The gravimetric capacitance of VOW-800 also reaches 317.8 F g^−1^, reflecting its superior energy storage capability (Figure 3e). The gravimetric capacitance of VOW-800 compared with reported V-based electrodes indicates that VOW-800 exhibits more prominent high gravimetric capacitance characteristics, offering broader application prospects (Table 1). This enhancement stems from the synergistic effect of the hierarchical pore structure and mixed-valence vanadium oxides (V^4+^/V^5+^), which collectively enhance ion diffusion and charge transfer. The interconnected mesoporous network significantly increases the electroactive area, allowing efficient electrolyte penetration and reducing ionic resistance. To investigate the effect of temperature on the graphitization degree of the carbon structure, Raman spectroscopy analysis was performed on the VOW-800 electrode (Figure 3f). The Raman spectrum shows two characteristic peaks: the D band (1350 cm^−1^), indicating the presence of disordered or defect-rich carbon structures, and the G band (1580 cm^−1^), corresponding to graphitic or sp^2^-hybridized carbon structures. The I_D_/I_G_ ratio of VOW-800 was calculated to be 0.94, suggesting a high degree of structural disorder within the carbon matrix, which plays a key role in enabling efficient charge transport through the interconnected network. The pristine balsa wood shows a smooth Raman profile without distinct peaks, indicating the absence of graphitic carbon structures (Figure S17). This confirms that thermal pyrolysis is essential for transforming wood into a conductive carbon matrix. The formation of a defect-rich conductive network in VOW-800 is critical for maintaining high conductivity and efficient charge transfer. Therefore, the 800 °C treatment is identified as the optimal condition, as it generates a defect-rich carbon network while maintaining sufficient structural integrity, which synergistically enhances capacitance and long-term stability.

Additionally, to characterize the active material content of the composite materials, thermogravimetric analysis (TG) was conducted under an oxygen atmosphere at temperatures ranging from 50 to 700 °C to determine the active material content of the electrodes (Figure S18). The purity of the carbon electrode was 98.03%, while the active mass of VO*_x_* in the V0W-600, 700, 800, and 900 composite material was about 48.03%, 42.15%, 37.32%, and 16.56%, respectively. Samples prepared by thermal cracking under a nitrogen atmosphere undergo gradual decomposition of vanadium oxides as the temperature increases, converting V^5+^ to V^4+^, resulting in a decrease in active mass with increasing processing temperature within the same test temperature range in thermal gravimetric analysis (TG) results under an oxygen atmosphere.

### 3.3. Electrochemical Performance Testing of VO_x_@Wood at 800 °C

**Figure 4:**
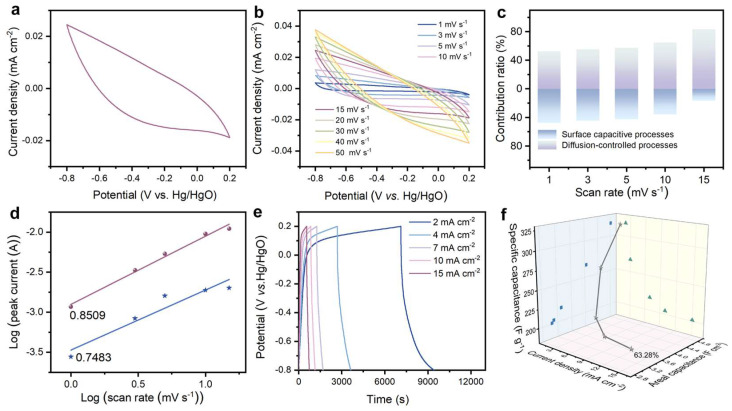
Electrochemical properties of VOW-800. (**a**) CV curve of VOW-800 at 15 mV s^−1^, (**b**) CV curves of VOW-800 at 1–50 mV s^−1^, (**c**) comparison of capacitance contribution of VOW-800 at 1–15 mV s^−1^ scan rate, (**d**) b-value analysis, (**e**) GCD curve of VOW-800 at 2–15 mA cm^−2^, (**f**) rate capability of VOW-800 at 2–15 mA cm^−2^.

The CV curve of VOW-800 at a scan rate of 15 mV s^−1^ shows no distinct redox peaks, indicating that the charge storage is dominated by electric double-layer capacitance rather than Faradaic pseudocapacitance (Figure 4a). To assess the electrochemical behavior of VOW-800, a series of electrochemical measurements were performed. The CV curves obtained at various scan rates (1 to 50 mV s^−1^) demonstrate broad and symmetric shapes, indicating good capacitive behavior and stability (Figure 4b). As the scan rate increases, the surface capacitive contribution becomes more dominant, indicating a faster ion adsorption/desorption process on the electrode surface (Figure 4c). To further understand the charge storage mechanism, kinetic analysis was performed using the CV data. The relationship between the current (I) and scan rate (v) follows the equation:

*I = av^b^* (1)

*I = k_1_v + k_2_v^1/2^* (2)

where ‘*a*’ and ‘*b*’ are modifiable parameters. The diffusion-controlled process exhibits current scaling with the square root of the scan rate, yielding a ‘*b*’ value of 0.5. In contrast, the capacitive process exhibits a linear relationship between current and scan rate, with ‘*b*’ set to 1. For VOW-800, the calculated *b* values are 0.75 and 0.85, indicating a combination of surface-controlled capacitance and diffusion-controlled processes (Figure 4d).

The GCD tests at varying current densities (2 to 15 mA cm^−2^) reveal that VOW-800 exhibits symmetric and stable profiles, indicating good reversibility and low internal resistance (Figure 4e). VOW-800 retains 63.28% of its areal capacitance when the current density increases from 2 to 15 mA cm^−2^, demonstrating excellent rate capability, which can be attributed to the well-developed porous network that reduces ion transport resistance (Figure 4f). The combination of a well-developed porous architecture and defect-engineered carbon framework endows VOW-800 with excellent electrochemical stability and high-rate performance, making it a promising candidate for high-performance supercapacitor applications. To characterize the cycling properties of the electrode material, the VOW-800 electrode was subjected to 10,000 cycles at 40 mA cm^−2^ (Figure S19), revealing a capacitance retention rate of 99.1%, providing new insights into structural optimization of VO*_x_* materials for energy storage applications.

**Figure 5:**
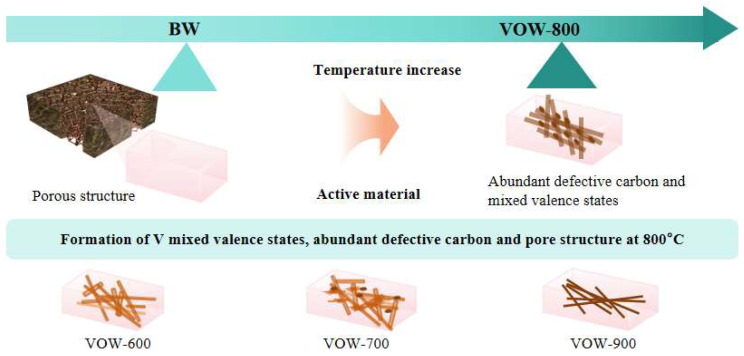
Schematic diagram of synergistic mechanisms.

Therefore, precise thermal modulation is crucial for preserving the structural integrity and ensuring the electrochemical stability of VO*_x_*/wood composites. A calcination temperature of 800 °C offers an optimal balance among electrical conductivity, surface area, and network stability, making it the most favorable condition for energy storage applications. Precise 800 °C heat treatment regulation forms abundant defects, which, combined with the porous structure formed after wood carbonization, creates more active sites in the activated carbon structure. This facilitates the adhesion of mixed-valence vanadium oxides and energy storage. The wood carbon structure and vanadium oxides jointly act as active materials in energy storage behavior. The three mechanisms collectively enhance the electrochemical performance of the electrode, enabling VO_x_@Wood to be applied in a broader range of electrochemical fields (Figure 5).

## 4. Conclusions

In conclusion, the large specific surface area and excellent structural compatibility of the wood-derived porous architecture significantly enhance both electron and ion transport. The successful integration of a variable-valent VO*_x_* heterostructure into the redox-active porous carbonized wood framework forms a conductive network that effectively addresses the inherent limitations of traditional electrode materials, such as sluggish electron transport and poor structural stability. The VO*_x_* aerogel network enables the specific surface area to reach 111.22 m^2^ g^−1^, and the surface specific capacitance of VOW-800 at 2 mA cm^−2^ is as high as 4.82 F cm^−2^. The VOW-800 electrode exhibits excellent energy storage capability and high capacitance characteristics, which provides new possibilities for the development of energy storage devices such as supercapacitors. This study not only broadens the application scope of wood in the field of energy storage, but also provides an important idea for the optimization and improvement of V_2_O_4_ electrode materials, which is of great significance in opening up potential avenues for promoting the development of green energy technology.

## Figures and Tables

**Table 1:** Comparison of the gravimetric capacitance of VOW-800 electrode material with conventional electrode materials.

Electrode Material	Gravimetric Capacitance	References
VOW-800	317.8 F g^−1^	
Monoclinic VO_2_(B) nanosheets	232.56 F g^−1^	[16]
Vanadium dioxide sulfur-doped reduced graphene oxide	204 F g^−1^	[17]
Coal slime-based activated carbon	220 F g^−1^	[53]
Nitrogen-doped agar-derived porous carbon(NAGC) electrode material	183 F g^−1^	[54]
Self-O-doped hierarchical porous carbon from yellow mangosteen fruit	217 F g^−1^	[55]
Poly(vinylidene fluoride)-derived carbon electrodes	249 F g^−1^	[56]

## Data Availability

Data are available upon request due to privacy or ethical restrictions. Data from this study are available from the corresponding authors upon request. Because of the privacy implications of the data in this study, these data are not publicly available.

## Supplementary Materials

The following supporting information can be downloaded at: https://www.mdpi.com/article/10.3390/nano15161249/s1. Figure S1: SEM image of the longitudinal section of BW; Figure S2: SEM image of the cross section of BW; Figure S3: The picture of V_2_O_5_@Wood; Figure S4: 3D schematic of the internal structure of VO*_x_*-600; Figure S5: 3D schematic of the internal structure of VO*_x_*-700; Figure S6: 3D schematic of the internal structure of VO*_x_*-900; Figure S7: X-ray diffraction patterns of VO_x_ electrodes and carbonised wood treated at different temperatures; Figure S8: The pore size distributions of BW; Figure S9: N2 adsorption/desorption curves of BW; Figure S10: The pore size distributions of VOW-600; Figure S11: N_2_ adsorption/desorption curves of VOW-600; Figure S12: The pore size distributions of VOW-700; Figure S13: N_2_ adsorption/desorption curves of VOW-700; Figure S14: The pore size distributions of VOW-900; Figure S15: N_2_ adsorption/desorption curves of VOW-900; Figure S16: Nyquist plot of VOW at different temperatures; Figure S17: Raman spectra of BW; Figure S18: Thermogravimetric (TG) testing of carbon electrodes, V_2_O_5_, and VOW-600, VOW-700, VOW-800, VOW-900; Figure S19: The VOW-800 electrode underwent 10,000 cycle tests at 40 mA cm^−2^; Table S1: Comparison of average pore size and specific surface area of electrodes under different treatment conditions.
