# Supplementary material for: Hierarchical VOx@Wood Aerogel Electrodes with Tunable Valence States for Enhanced Energy Storage
Source: Nanomaterials (Basel). 2025 Aug 14;15(16):1249. doi: 10.3390/nano15161249 (PMC12388124; doi:10.3390/nano15161249)
Supplement: Supplementary file 1 [file nanomaterials-15-01249-s001.zip › nanomaterials-3742120-supplementary.pdf]

Hierarchical VO_x@Wood Aerogel Electrodes with Tunable Valence States for Enhanced Energy Storage

Yu Wang [a], *Yuan Yu* [a], *Zhenle Hu* [a], *Lei Qiao* [a], *Huaiyuan Peng* [a], *Jingwen Xie* [a], *Haiyue Yang* [a*], *Chengyu Wang* [a*]

[a] Key Laboratory of Bio-Based Material Science and Technology of Ministry of Education, Northeast Forestry University, Harbin, Heilongjiang, 150040, China

[*] Corresponding author: haiyueyang@nefu.edu.cn(H.Yang)

[*] Corresponding author: wangcy@nefu.edu.cn (C. Wang)

Characterizations

To extensively examine the morphology of electrodes, a Field-Emission Scanning Electron Microscope (FE-SEM, Apreo S HiVac, Thermo Scientific, USA) was utilized. For a detailed analysis of physical structures and chemical properties. Elemental and chemical analyses were carried out using X-ray Photoelectron Spectroscopy (XPS), employing the K-Alpha (Thermo, USA). The Brunauer-Emmett-Teller (BET, JW-BK132F, JWGB, China) method was applied to determine the specific surface area and pore size distribution, based on nitrogen and air adsorption isotherms measured at -196 ℃. The carbon structure and its pair conductivity were analysed by Raman spectroscopy (HORIBA, LabRAM HR Evolution).An X-ray diffractometer (XRD, D/max 2200 VPC, Rigaku, Japan) was used to characterise the crystalline structure of the samples, with a scanning angle of 5° to 80° and a scanning speed of 4° min^{-1}.The thermal stability of the samples was studied using a thermogravimetric analyser (TGA, STA 6000-SQ8) to measure the thermal stability of the samples. The samples were heated from 50 ℃ to 900 ℃ at a rate of 10 ℃ min^{-1} in a oxygen atmosphere.

Electrochemical measurements

All electrochemical assessments were conducted on a CHI-660E electrochemical workstation. For the standard three-electrode setup, the VO_x@Wood served as the working electrode, Hg/HgO as the reference electrode, and a platinum plate as the counter electrode. The electrolyte consisted of 6.0 M KOH. Cyclic voltammetry (CV) tests were executed within the potential window of -0.8 to 0.2V (vs. Hg/HgO), encompassing various scan rates ranging from 1 to 50 mV s^{-1}. Galvanostatic charge-discharge (GCD) measurements were performed between -0.8 and 0.2 V (vs. Hg/HgO) at current densities spanning from 2 to 50 mA cm^{-2}. The areal capacitance of the electrode was calculated based on the following equation:

$$C_a = I_a t/\triangle U \quad \text{(S1)}$$

$$C_g = I_g t/\triangle U \quad \text{(S2)}$$

where C_a (F cm^{-2}), I_a (A cm^{-2}), t (s), $\triangle U$ (V), C_g (F g^{-1}) and I_g (A g^{-1}) represent the areal capacitance, the areal current density, the discharge time, the potential window, the gravimetric capacitance and the gravimetric current density of the discharge process, respectively.

Supplementary figures

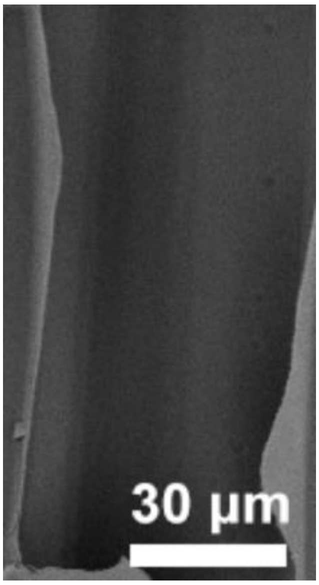

Figure S1.SEM image of the longitudinal section of BW

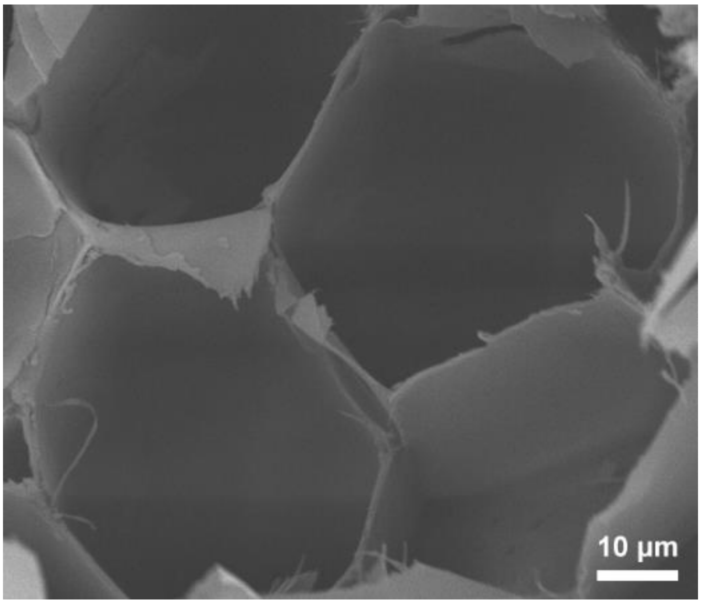

Figure S2.SEM image of the cross section of BW

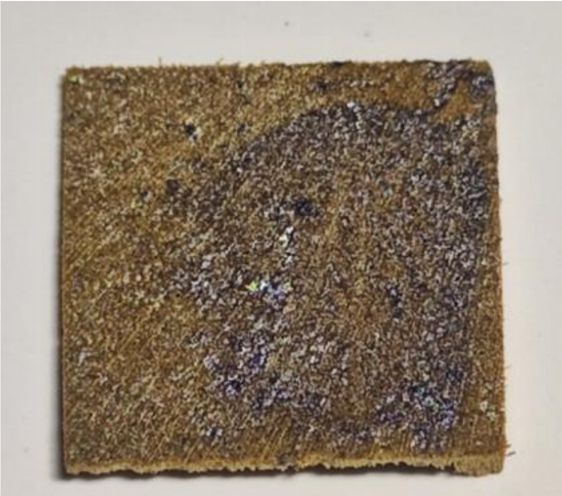

.

Figure S3. The picture of V_2O_5@Wood.

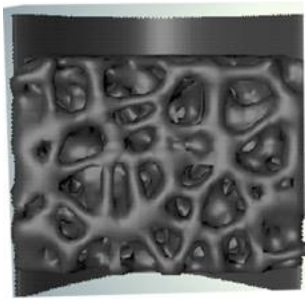

Figure S4. 3D schematic of the internal structure of VO_x-600.

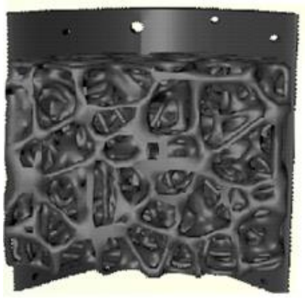

Figure S5. 3D schematic of the internal structure of VO_x-700.

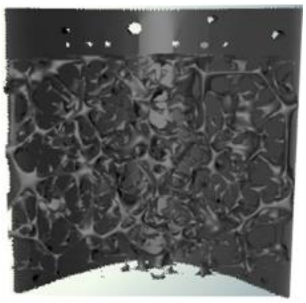

Figure S6. 3D schematic of the internal structure of VO_x-900.

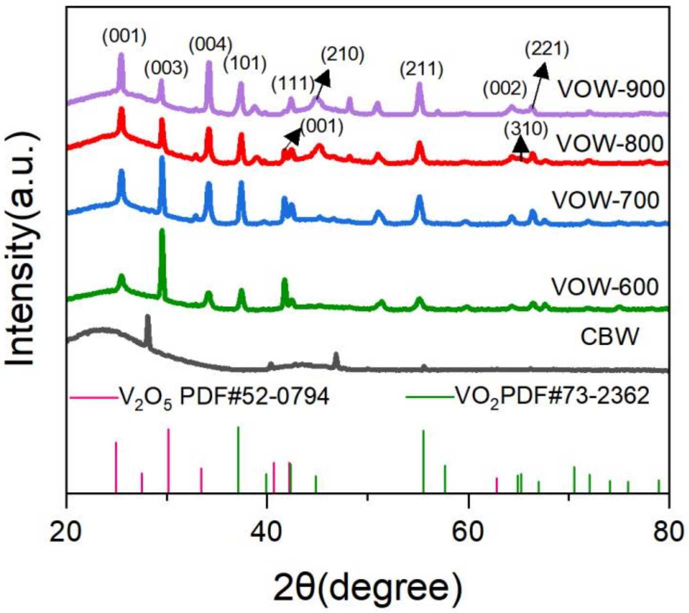

Figure S7.X-ray diffraction patterns of VO_x electrodes and carbonised wood treated at different temperatures.

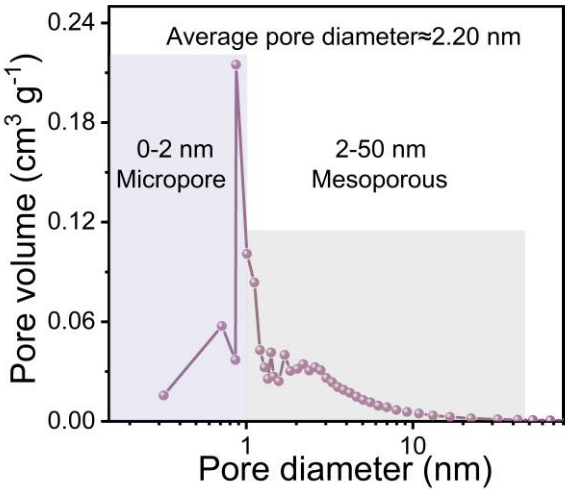

Figure S8. The pore size distributions of BW.

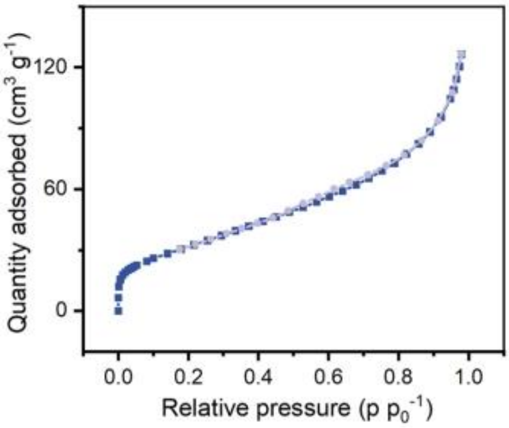

Figure S9. N_2 adsorption/desorption curves of BW.

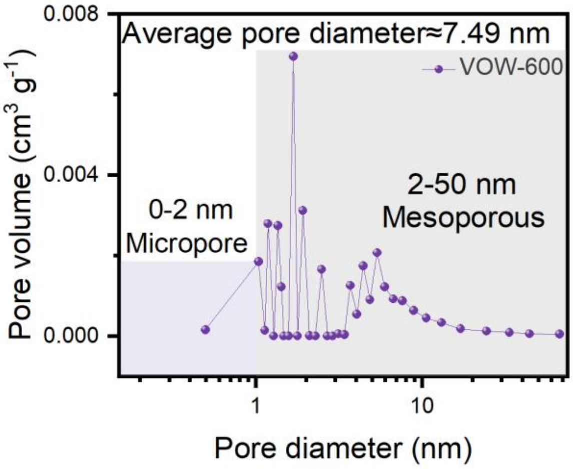

Figure S10. The pore size distributions of VOW-600.

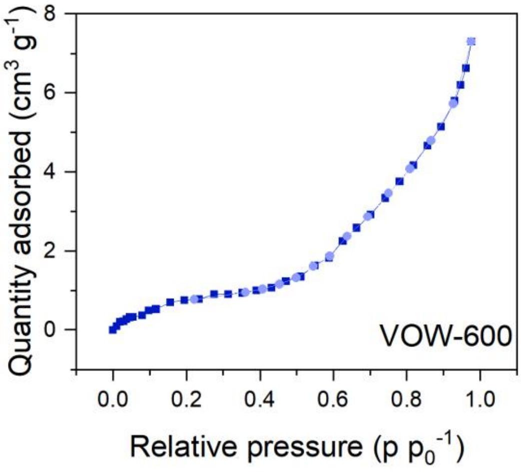

Figure S11. N_2 adsorption/desorption curves of VOW-600.

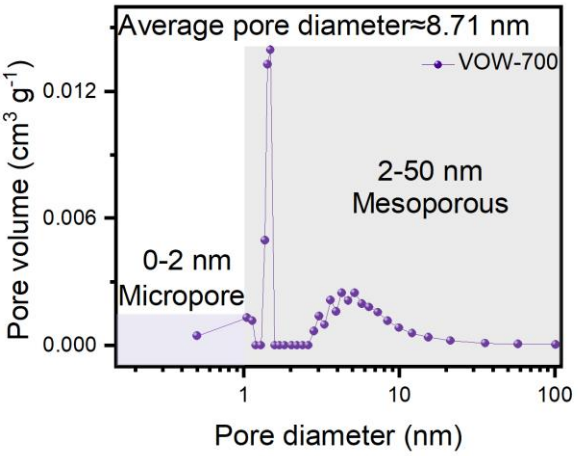

Figure S12. The pore size distributions of VOW-700.

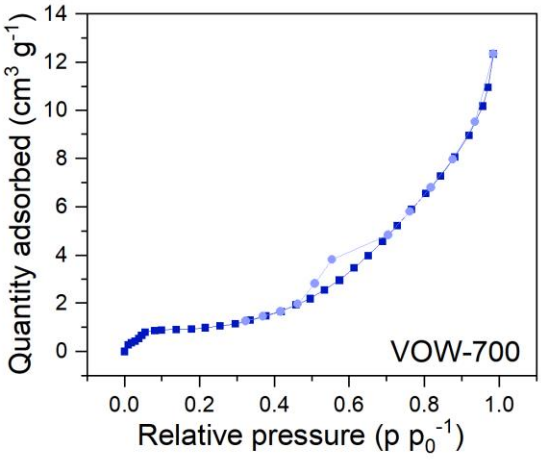

Figure S13. N_2 adsorption/desorption curves of VOW-700.

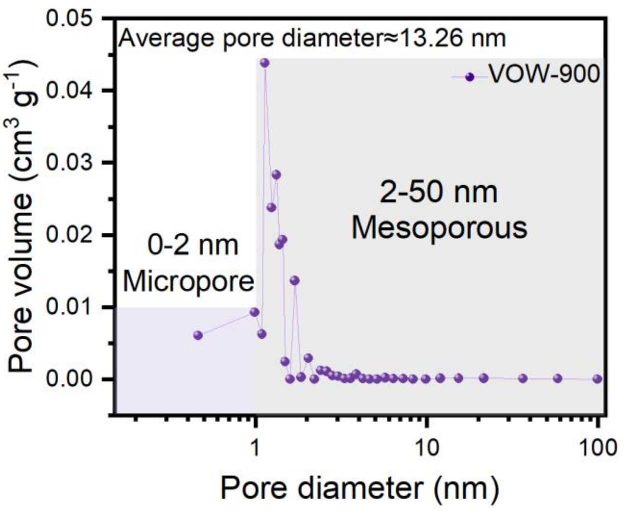

Figure S14. The pore size distributions of VOW-900.

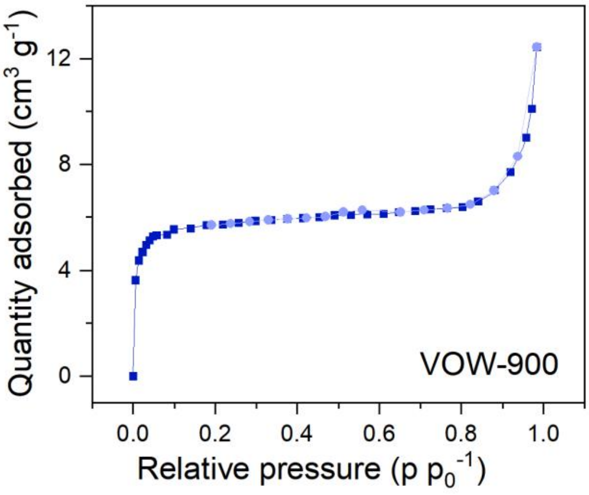

Figure S15. N_2 adsorption/desorption curves of VOW-900.

Table S1. Comparison of average pore size and specific surface area of electrodes under different treatment conditions

Electrode	Average pore diameter (nm)	Specific surface area（$m^2\ g^{-1}$）
BW	2.20	3.96
VOW-600	7.49	3.32
VOW-700	8.71	3.85
VOW-800	5.11	111.2
VOW-900	13.26	20.7

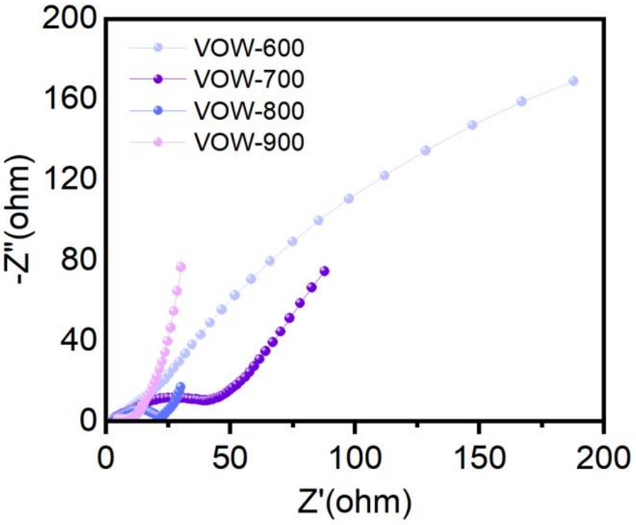

Figure S16. Nyquist plot of VOW at different temperatures

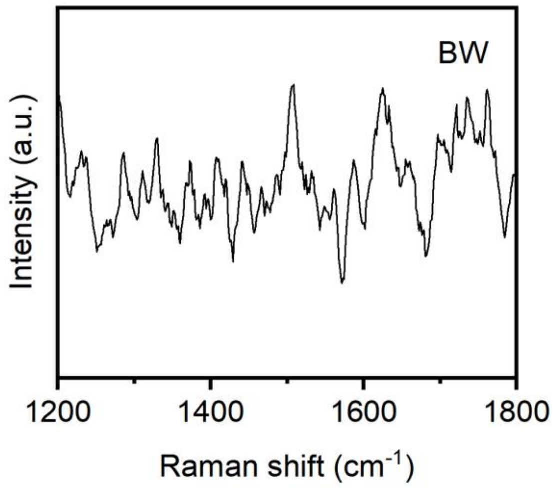

Figure S17. Raman spectra of BW.

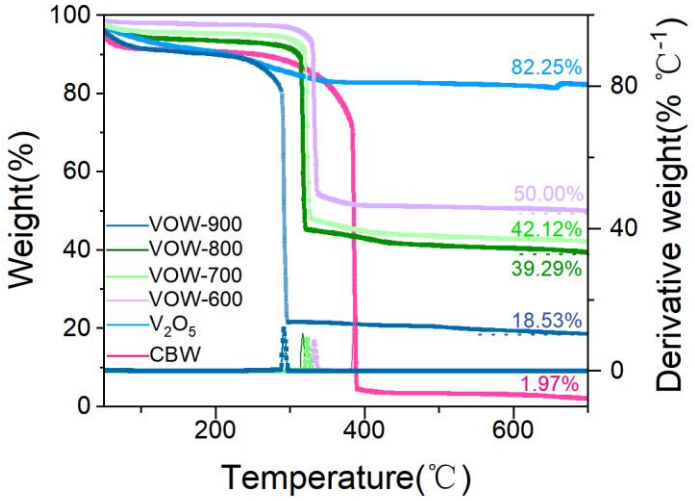

Figure S18. Thermogravimetric (TG) testing of carbon electrodes, V_2O_5, and VOW-600，VOW-700,VOW-800,VOW-900.

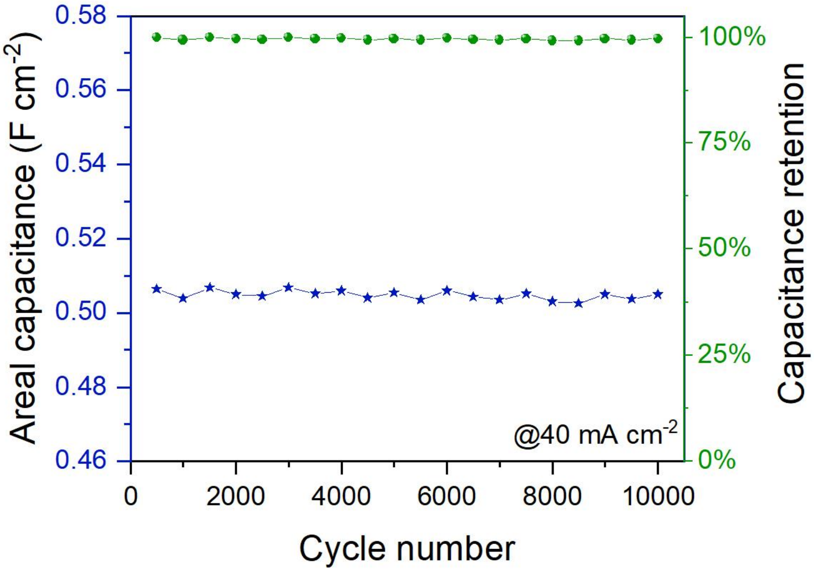

Figure S19. The VOW-800 electrode underwent 10,000 cycle tests at 40 mA cm^{-2}**.**
